# Maternal Hemoglobin Concentrations and Birth Weight, Low Birth Weight (LBW), and Small for Gestational Age (SGA): Findings from a Prospective Study in Northwest China

**DOI:** 10.3390/nu14040858

**Published:** 2022-02-18

**Authors:** Danmeng Liu, Shanshan Li, Binyan Zhang, Yijun Kang, Yue Cheng, Lingxia Zeng, Fangyao Chen, Baibing Mi, Pengfei Qu, Doudou Zhao, Zhonghai Zhu, Hong Yan, Duolao Wang, Shaonong Dang

**Affiliations:** 1 Department of Epidemiology and Health Statistics, School of Public Health, Health Science Center, Xi’an Jiaotong University, Xi’an 710061, China; liudanmeng1214@stu.xjtu.edu.cn (D.L.); zhangbinyan@stu.xjtu.edu.cn (B.Z.); tjkyj@xjtu.edu.cn (Y.K.); tjzlx@mail.xjtu.edu.cn (L.Z.); chenfy@xjtu.edu.cn (F.C.); xjtu.mi@xjtu.edu.cn (B.M.); zhonghai_zhu@mail.xjtu.edu.cn (Z.Z.); yanhonge@xjtu.edu.cn (H.Y.); 2 School of Public Health, Shandong First Medical University & Shandong Academy of Medical Sciences, Taian 271016, China; lishanshan@sdfmu.edu.cn; 3 Department of Nutrition, School of Public Health, Health Science Center, Xi’an Jiaotong University, Xi’an 710061, China; chengy@mail.xjtu.edu.cn; 4 Translational Medicine Center, Northwest Women’s and Children’s Hospital, Xi’an 710061, China; xinxi3057@stu.xjtu.edu.cn (P.Q.); zhaodoudou1223@stu.xjtu.edu.cn (D.Z.); 5 Nutrition and Food Safety Engineering Research Center of Shaanxi Province, Xi’an 710061, China; 6 Key Laboratory of Environment and Gene-Related Diseases, Xi’an Jiaotong University, Ministry of Education, Xi’an 710061, China; 7 Department of Clinical Sciences, Liverpool School of Tropical Medicine, Liverpool L3 5QA, UK; duolao.wang@lstmed.ac.uk

**Keywords:** maternal hemoglobin concentration, neonatal birth weight, LBW, SGA, nonlinear association, prospective study

## Abstract

Birth weight and related outcomes have profound influences on life cycle health, but the effect of maternal hemoglobin concentration during pregnancy on birth weight is still unclear. This study aims to reveal the associations between maternal hemoglobin concentrations in different trimesters of pregnancy and neonatal birth weight, LBW, and SGA. This was a prospective study based on a cluster-randomized controlled trial conducted from July 2015 to December 2019 in rural areas of Northwest China. Information on maternal socio-demographic status, health-related factors, antenatal visits, and neonatal birth outcomes were collected. A total of 3748 women and their babies were included in the final analysis. A total of 65.1% and 46.3% of the participants had anemia or hemoglobin ≥ 130 g/L during pregnancy. In the third trimester, maternal hemoglobin concentration was associated with birth weight in an inverted U-shaped curve and with the risks of LBW and SGA in extended U-shaped curves. The relatively higher birth weight and lower risks for LBW and SGA were observed when hemoglobin concentration was 100–110 g/L. When maternal hemoglobin was <70 g/L or >130 g/L, the neonatal birth weight was more than 100 g lower than that when the maternal hemoglobin was 100 g/L. In conclusion, both low and high hemoglobin concentrations in the third trimester could be adverse to fetal weight growth and increase the risks of LBW and SGA, respectively. In addition to severe anemia, maternal hemoglobin >130 g/L in the third trimester should be paid great attention to in the practice of maternal and child health care.

## 1. Introduction

Birth weight is critical for the evaluation of fetal growth and the prediction of neonatal mortality and morbidity [1]. The poor birth weight caused by limited intrauterine growth and development could further result in adverse birth outcomes including low birth weight (LBW) and small for gestational age (SGA). According to the WHO criteria, LBW infants are those born weighing < 2500 g [2], and infants born SGA are defined as those weighing below the 10th centile of birth weight by sex for a specific completed gestational age of a given reference population [3]. The prevalence of LBW/SGA is relatively high in the middle- and low-income countries, especially in economically undeveloped regions [3,4]. Both LBW and SGA are the main causes of neonatal mortality and morbidity. Infants with LBW/SGA have a considerably higher risk of short-term morbidities, including infections, respiratory depression, jaundice, hypoglycemia, hypothermia, and so on. In the long-term, LBW/SGA are related to many health issues, including cognitive deficiencies, mental retardation, cerebral palsy, gastrointestinal morbidity, and metabolic disorders such as obesity, diabetes, and cardiovascular diseases [2,4,5,6]. Therefore, poor fetal weight growth harms life cycle health, and identifying its potential influencing factors is of great significance for the prevention of short- and long-term diseases.

Maternal nutrition during pregnancy critically determines fetal nutrition and has a significant contribution to fetal and neonatal health. Maternal intakes of micronutrients such as folic acid and iron are crucial for fetal growth and development [7]. Hemoglobin concentration is a key indicator reflecting the maternal nutrition status during pregnancy, especially the iron status [8]. Previous studies reported that maternal hemoglobin concentration was associated with neonatal birth weight as well as LBW and SGA, but there is no consistent conclusion. Rasmussen et al. found a strong independent inverse correlation between the lowest second-trimester hemoglobin and birth weight, but no relationship was observed in the first trimester [9]. Haider et al. reported that neonatal birth weight increased by 14 g for every 1 g/L increase in average hemoglobin in the third trimester. Steer et al. observed that the maximum mean birth weight was achieved with a lowest hemoglobin concentration in pregnancy of 85–95 g/L, which indicated a nonlinear relationship between maternal hemoglobin during pregnancy and birth weight [10]. A recent meta-analysis revealed a U-shaped curve association between maternal hemoglobin concentration and adverse birth outcomes, which suggested that both low and high hemoglobin concentrations might be risk factors for fetal growth [11].

Pregnant women have a high prevalence of anemia [12]. Lots of studies have reported that maternal anemia during pregnancy is associated with risks of intrauterine growth restriction, preterm birth, LBW, and SGA [13,14,15]. However, the associations were diverse in different gestational trimesters. The associations between maternal anemia and the increased risks of preterm birth, LBW, and SGA were strong in the first trimester [16,17,18,19], but were weak in the second or third trimester [16,18,20,21,22,23]. Additionally, maternal anemia in the third trimester may reduce the risks of adverse birth outcomes [21]. As for the associations between high hemoglobin concentration and birth outcomes, the results in the existing literature are controversial. Some studies showed that high hemoglobin concentration significantly reduced the risks of LBW or preterm birth [23,24]. However, several studies indicated that maternal higher hemoglobin concentrations during pregnancy were associated with increased risks of adverse birth outcomes [18,22,25].

Accordingly, the relationships between maternal hemoglobin concentration and birth outcomes reported in previous studies are complicated and have been disputed. The inconsistencies of the findings were mainly attributed to the differences in research design, sample size, time of hemoglobin measurement, hemoglobin classification cut-offs, race, and region of investigation [20,23]. Additionally, most of the previous studies focused on the effect of maternal anemia, and the relatively fewer studies that reported the association of maternal high hemoglobin concentrations with birth outcomes were conducted mainly in developed countries [26]. Evidence from the Chinese population, especially those in the underdeveloped regions, is lacking. Therefore, the present study aims to reveal the associations between maternal hemoglobin concentrations (within the entire hemoglobin range) in different trimesters of pregnancy and neonatal birth weight, LBW, and SGA, through a prospective study in Northwest China.

## 2. Materials and Methods

### 2.1. Study Design and Participants

The data was from a cluster-randomized controlled trial (registration number in ClinicalTrials.gov: NCT02537392) which was conducted from July 2015 to December 2019 in rural areas of Northwest China. The original trial aimed to evaluate the effect of micronutrient supplementation during pregnancy on congenital heart disease (primary outcome) and other birth outcomes. Detailed design and methods of this trial have been described elsewhere [27]. A brief introduction is as follows. A cluster randomization method was used to randomize the townships (the unit of randomization) of the study region to the intervention groups (the folic acid group (the control group), folic acid + iron group, and folic acid + vitamin B complex group) with a 1:1:1 ratio before enrolment. Women aged 15–49 years and with less than 20 weeks gestational age were invited to participate, and the exclusion criteria at recruitment included (1) use of supplements containing iron or vitamin B complex for more than 2 weeks, (2) having given birth to children with congenital heart disease or other birth defects, and (3) having severe liver or kidney disease. In each township, eligible women with informed written consent were enrolled at the township-level health center, administered by a trained township maternal and child healthcare professional. Participants in each intervention group were required to use the corresponding nutrient supplements daily until delivery and were followed up from the time of enrolment to delivery. Information on maternal socio-demographic status, health-related factors, antenatal visits, and neonatal birth outcomes was collected. The participants received routine treatment and health care according to the Chinese clinical guidelines for the diagnosis and treatment of maternal anemia if they developed this during pregnancy [28].

**Figure 1:**
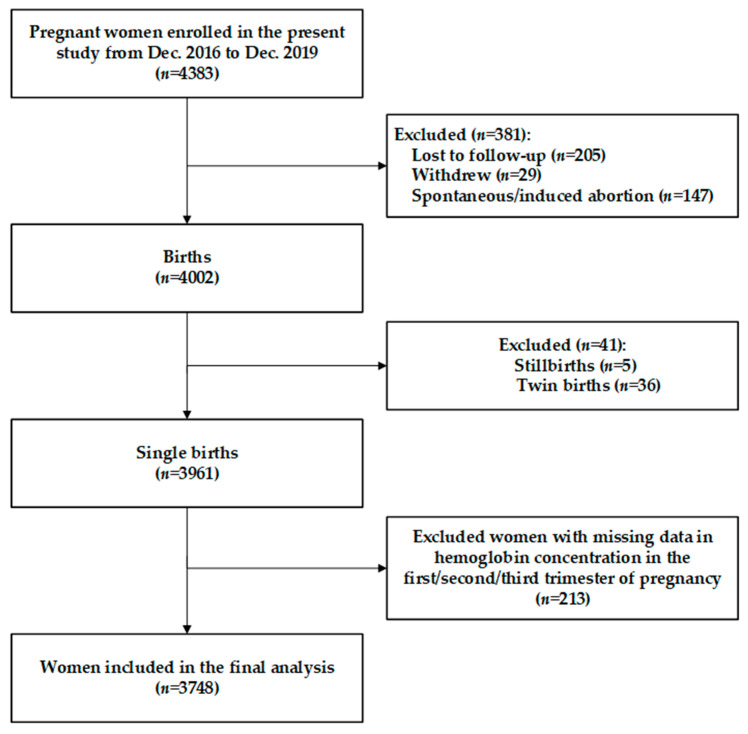
Study flowchart.

Based on the original trial, the present study was conducted to investigate the associations between maternal hemoglobin during pregnancy and birth weight, LBW, and SGA. A total of 4383 eligible pregnant women enrolled in the original trial from December 2016 to December 2019 were included in the present study. Women who were lost to follow-up (n 205), withdrew (n 29), had spontaneous/induced abortion (n 147), had a stillbirth (n 5) or twin births (n 36) were excluded. In addition, since we focused on the maternal hemoglobin during pregnancy according to the study objective, a total of 3748 women who had hemoglobin records in each of the gestational trimesters (including the first, second, and third trimester) were finally included in the study (Figure 1). Women included in the final analysis had no significant difference in maternal socio-demographic characteristics and parity compared to those excluded from the study (Table S1).

### 2.2. Sample Size

The sample size was calculated based on the main outcome (birth weight) of the study. Estimating the mean and standard deviation of the neonatal birth weight of women with non-anemia during pregnancy was 3250 g and 440 g [29], assuming the mean neonatal birth weight of anemic mothers was 50 g lower than the nonanemic mothers, and using a two-sided significance level of 5% and a power of 80%, a minimum estimated sample size was 1216 in each group. Considering a 10% loss rate, a minimum total of 2676 (1338 in each group) pregnant women were required. Our study included 3748 women in the final analysis, which met the sample size requirement.

### 2.3. Data Collection

Participants enrolment and follow-ups were conducted in the township-level health centers, and deliveries were completed in the county-level hospitals. Data collection was carried out by trained township maternal and child healthcare professionals and was supervised by investigators from Xi’an Jiaotong University Health Science Center. At enrolment, maternal socio-demographic characteristics (including age, education, occupation, and income), reproductive history, and date of last menstrual period were collected via face-to-face interviews. Baseline height and weight were measured using unified facilities. During follow-ups, after women attended the antenatal visits at county-level hospitals, the township maternal and child healthcare professionals collected their anthropometric and hematological records from their antenatal visits and accounted the number of antenatal visits at the end of the follow-ups. Maternal uses of interventional micronutrient supplements were investigated every two months during follow-ups, and other maternal micronutrient supplementations during pregnancy were collected within one week after delivery. Neonatal birth outcomes, including birth date, birth weight, gestational age, and gender, were obtained according to medical records. All information was entered into a web-based surveillance system by trained township maternal and child healthcare professionals, and routine data quality monitoring was carried out by researchers from Xi’an Jiaotong University Health Science Center.

### 2.4. Hemoglobin Measurement

Maternal hemoglobin measurement was conducted during antenatal visits at the county-level hospitals by the clinical laboratory technician. Hemoglobin concentration in a unit of g/L was measured through routine blood tests which were completed using the automatic hematology analyzer with the venous blood samples of participants. The township maternal and child healthcare professionals recorded the hemoglobin concentration according to the test results after antenatal visits.

The gestational age at antenatal visits was calculated according to the last menstrual period. The period of pregnancy was divided into trimesters according to the gestational age, which included the first trimester (0–12 weeks of gestational age), the second trimester (13–27 weeks of gestational age), and the third trimester (28 weeks of gestational age to the time of delivery). The present study only included women with at least one hemoglobin record in each trimester, and for women who had more than one hemoglobin record in a certain trimester, the lowest was used for analysis [10,30].

Maternal hemoglobin concentration < 110 g/L can be diagnosed as gestational anemia according to the WHO standard, and the severity of anemia was defined as follows: 100–109 g/L is mild anemia, 70–99 g/L is moderate anemia, and <70 g/L is severe anemia [31]. Maternal hemoglobin ≥ 130 g/L referred to a relatively-high hemoglobin concentration during pregnancy [24,26,32].

### 2.5. Outcome Assessment

The primary outcome of this study was neonatal birth weight, and the secondary outcomes were LBW and SGA. Birth weight was measured to the nearest 10 g with a baby scale within one hour after delivery. Gender (male/female) was recorded after delivery. Gestational age at delivery was calculated according to the last menstrual period and was confirmed by ultrasound scans. LBW was defined as birth weight < 2500 g according to the WHO [2]. SGA was defined as birth weight below the 10th percentile for gestational age and sex according to the standards for fetal growth and development in China [33].

### 2.6. Covariate Assessment

Based on the existing literature [4,34], covariates considered in the study mainly included two parts: (1) socio-demographic characteristics, including maternal age at enrolment (<25/25–34/≥35 years), education level (junior high school or below/senior high school/college or above), occupation (farmers/others), per capita annual household income (<5000/5000–9999/≥10,000 Yuan, where 1 Yuan = 0·156 $US on 8 November 2021), and township (48 townships); (2) health-related characteristics, including parity (primipara/multipara), gestational age at enrolment (≤12 weeks/>12 weeks), body mass index (BMI) at enrolment (underweight < 18.5/normal weight 18.5–23.9/overweight 24.0–27.9/obesity ≥ 28.0 kg/m^2^), the times of antenatal visits (≤5/>5), and micronutrient supplementations (folic acid/folic acid + iron/folic acid + vitamin B complex). Maternal age and gestational age at enrolment were respectively calculated according to birth date and the last menstrual period. Height was measured to the nearest 0.1 cm with a stadiometer, and weight was measured to the nearest 0.1 kg with an electronic scale. BMI at enrolment was calculated as weight at enrolment in kilograms divided by height in meters squared, and was classified according to the standards recommended in the “Guidelines for prevention and control of overweight and obesity in Chinese adults” [35]. Micronutrient supplementation was classified according to the intervention groups in the original trial. 

### 2.7. Statistical Analysis

Continuous variables were expressed as mean ± SDs, and categorical variables were described as numbers (proportions). Since the sample of the present study was obtained based on a cluster-randomized controlled trial (the township is the unit of randomization), the data collected had a hierarchical structure. Accordingly, generalized estimating equation (GEE) models [36] were applied to evaluate the associations of maternal hemoglobin concentration during pregnancy with the outcomes. The GEE model is a population average model used to estimate the associations between neighborhood characteristics (this refers to the factors related to the neighborhood that the study individuals belong to, and possess both physical and social attributes that could plausibly affect the health of individuals [37]) and health outcomes in multilevel studies [36]. In this study, the variable of township had the random effect, and maternal hemoglobin concentration in different trimesters of pregnancy and covariates had the fixed effects in the GEE models. The unadjusted model and adjusted model were established to estimate the changes in birth weight (normal distribution and identity link function) and RR for LBW/SGA (binomial distribution and log link function), as well as their accompanying 95% CI. The adjusted model was adjusted for socio-demographic characteristics (including maternal age, education, occupation, and per capita annual household income) and health-related characteristics (including parity, BMI at enrolment, gestational age at enrolment, number of antenatal visits, and micronutrient supplementation). When estimating the associations between maternal hemoglobin and birth weight/LBW, models were additionally adjusted for neonatal gender and gestational age at delivery.

Restricted cubic spline (RCS) function was further used to estimate the dose–response relationship between maternal hemoglobin concentration in different trimesters of pregnancy and the birth outcomes [38]. According to the cut-offs of hemoglobin that were used to classify the severity of gestational anemia [31] and the cut-off that represents a relatively-high maternal hemoglobin concentration [20,24], maternal hemoglobin at 70, 100, 110, 130 g/L were selected as the four knots set into the RCS models. In these models, maternal hemoglobin in different trimesters was set as independent variable (x-axis), and birth weight/LBW/SGA was set as dependent variable (y-axis). Hemoglobin at 110 g/L or the minimum value of hemoglobin was set as the reference value. Models were adjusted for socio-demographic and health-related characteristics as described above. The *p*-value for the overall association was used to evaluate the overall association between maternal hemoglobin and birth outcomes, and the *p*-value for the nonlinear association was used to assess any nonlinear association between maternal hemoglobin and birth outcomes.

Based on the above analyses, subgroup analyses according to maternal hemoglobin concentration and maternal characteristics (age, education level, income level, parity, BMI at enrolment, times of antenatal visits, and micronutrient supplementation) were further conducted to estimate the associations between maternal hemoglobin during pregnancy and neonatal birth weight in different subgroups.

All analyses were performed using SAS version 9.4 (SAS Institute, Cary, NC, USA). All statistical tests were two-tailed, and statistical significance was set as *p* < 0.05. SAS code for GEE models could be accessed in “SAS System Documentation” and referred to the GEE syntax under “The GENMOD Procedure”. The RCS curve fitting was realized by an SAS macro program written by Desquilbet [38].

## 3. Results

### 3.1. Maternal Baseline Characteristics and Neonatal Birth Outcomes

As shown in Table 1, higher proportions of participants were observed in women aged 25–34 years (59.6%), with junior high school or below educational level (54.4%), farmers (87.8%), medium per capita annual household income (40.5%), normal weight (71.0%), and having attended no more than five antenatal visits (67.2%). 

The average neonatal birth weight was 3233.4 ± 418.3 g, and the average gestational age at delivery was 39.7 ± 1.3 weeks. The proportions of LBW and SGA infants were 2.6%, and 13.4%, respectively.

### 3.2. Maternal Hemoglobin Status in Different Trimesters of Pregnancy

Maternal hemoglobin concentrations in the first, second, and third trimesters of pregnancy were tested averagely at 10.2 (SD 2.0), 19.4 (SD 3.2), and 36.1 (SD 2.7) weeks of gestation. As displayed in Table 2, in the first, second, and third trimester, the prevalence of maternal anemia was 16.6%, 30.9%, and 45.9%; the rates of hemoglobin ≥130 g/L were 33.1%, 14.7%, and 8.2%, respectively. A total of 65.1% and 46.3% of the participants have had anemia or hemoglobin ≥130 g/L during pregnancy.

### 3.3. Associations between Maternal Hemoglobin Concentrations during Pregnancy and Birth Weight-Related Outcomes

Table 3 displays the associations between maternal hemoglobin concentrations in different trimesters and neonatal birth weight, LBW, and SGA. In the first trimester, compared to women with normal hemoglobin concentration (110–129 g/L), women with hemoglobin ≥ 130 g/L had a significant increase in neonatal birth weight (adjusted changes: 26.5 g, 95% CI: 0.2, 52.8); no association between maternal hemoglobin with LBW and SGA was observed. In the second trimester, no association was found between maternal hemoglobin and the outcomes. In the third trimester, compared to women with normal hemoglobin concentration (110–129 g/L), women with severe anemia (<70 g/L) had a significant decrease in neonatal birth weight (adjusted changes: −216.3 g, 95% CI: −426.7, −5.9), and had a significant increase in risk of LBW (adjusted RR: 7.47, 95% CI: 2.53, 22.08). Women with mild anemia (100–109 g/L) had a significant increase in neonatal birth weight (adjusted changes: 46.5 g, 95% CI: 7.6, 85.3), and had a 27% reduced risk of SGA (adjusted RR: 0.73, 95% CI: 0.61, 0.87). Women with hemoglobin ≥130 g/L had a trend of reduction in neonatal birth weight, but the result was not statistically significant (adjusted changes: −30.7 g, 95% CI: −76.1, 14.7).

### 3.4. Dose–Response Relationships between Maternal Hemoglobin Concentrations and Birth Weight-Related Outcomes

**Figure 2:**
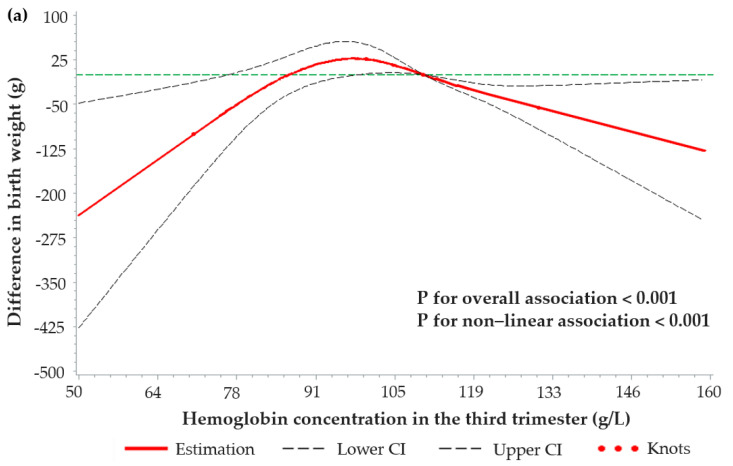

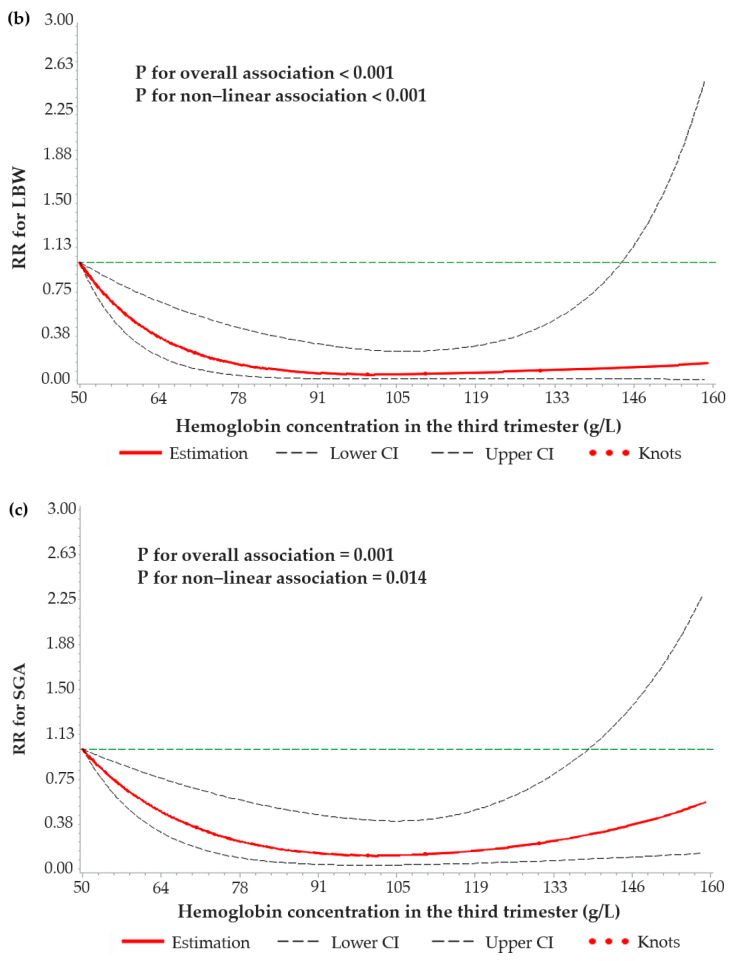
Dose-response relationships between maternal hemoglobin concentrations in the third trimester and (**a**) birth weight, (**b**) LBW, and (**c**) SGA. The associations were estimated using restricted cubic spline functions with four knots (including hemoglobin at 70, 100, 110, 130 g/L). Models were adjusted for socio-demographic characteristics (including maternal age, education, occupation, and per capita annual household income) and health-related characteristics (including parity, BMI at enrolment, gestational age at enrolment, number of antenatal visits, and micronutrient supplementation). When estimating the associations between maternal hemoglobin concentrations and birth weight/LBW, models were additionally adjusted for neonatal gender and gestational age at delivery. For birth weight, hemoglobin at 110 g/L was set as the reference value, and for LBW and SGA, the minimum value of hemoglobin was set as the reference value. Dashed lines represent the 95% CIs, and knots were displayed by dots. The horizontal dashed green line represents whether the difference in birth weight was 0 g or the RR for LBW/SGA was 1.00.

RCS functions with four knots (maternal hemoglobin at 70, 100, 110, 130 g/L) were further applied to estimate the dose–response relationships between maternal hemoglobin in different trimesters of pregnancy and birth outcomes. In the first and second trimester, no dose–response relationship was found (data was not shown). In the third trimester, dose–response relationships were observed between maternal hemoglobin concentration and neonatal birth weight, LBW, and SGA (Figure 2).

An inverted U–shaped curve was fitted between maternal hemoglobin concentration in the third trimester and neonatal birth weight (both *p*-values for overall and non-linear association were <0.001). The relatively-higher neonatal birth weight was obtained among women with a hemoglobin concentration of close to 100 g/L. When maternal hemoglobin was <70 g/L, neonatal birth weight significantly increased with the increase of hemoglobin; when maternal hemoglobin was 70–100 g/L, birth weight still showed an increasing trend when maternal hemoglobin was rising; when maternal hemoglobin was >100 g/L, neonatal birth weight significantly decreased with the increase of maternal hemoglobin; when maternal hemoglobin was >130 g/L, neonatal birth weight was more than 100 g lower than that when the maternal hemoglobin was 100 g/L (Figure 2a).

Extended U-shaped curves were obtained between maternal hemoglobin concentration in the third trimester with the risks of LBW and SGA (both *p*-values for overall and non-linear association were <0.05). Both the relatively lower risks for LBW and SGA were obtained among women with a hemoglobin concentration of 100–110 g/L. When maternal hemoglobin was <100 g/L, risks of both LBW and SGA were sharply increased with the decrease of hemoglobin; when maternal hemoglobin was >110 g/L, the risk of LBW was slightly increased with the rise of hemoglobin, and the risk of SGA was significantly increased with the increase of hemoglobin (Figure 2b,c).

### 3.5. Associations between Maternal Hemoglonbin in the Third Trimester and Birth Weight-Related Outcomes According to Hemoglobin Concentration and Maternal Characteristics

According to the above result, we used hemoglobin of 100 g/L in the third trimester as the cut-off to divide women into two groups and conducted the subgroup analyses according to maternal characteristics in each group. In the third trimester, among women with hemoglobin < 100 g/L, there is a positive but non-significant association between maternal hemoglobin and neonatal birth weight (adjusted changes: 2.4 g, 95% CI: −0.5, 5.4). Among women with hemoglobin ≥100 g/L, maternal hemoglobin was significantly negatively associated with neonatal birth weight (adjusted changes: −2.6 g, 95% CI: −4.1, −1.0). Similar associations were found in most of the subgroups according to maternal characteristics (Table S2). 

In the third trimester, among women with hemoglobin <100 g/L, maternal hemoglobin was negatively associated with the risks of LBW/SGA (LBW: adjusted RR: 0.58, 95% CI: 0.42, 0.79; SGA: adjusted RR: 0.81, 95% CI: 0.67, 0.98). Among women with hemoglobin ≥100 g/L, maternal hemoglobin was positively associated with the risk of SGA (adjusted RR: 1.13, 95% CI: 1.04, 1.23). Similar associations were found in many subgroups according to maternal characteristics (Table S3).

## 4. Discussion

In the present prospective study, about two-thirds of the participants had anemia during pregnancy, and about half of the participants had hemoglobin ≥ 130 during gestation. We found that maternal hemoglobin concentration in the third trimester had an inverted U-shaped association with neonatal birth weight, and had extended U-shaped associations with the risks of LBW and SGA. The relatively higher birth weight and lower risks for LBW and SGA were observed when hemoglobin concentration was 100–110 g/L. Maternal hemoglobin < 70 g/L or >130 g/L was strongly related to the decreased birth weight and increased risks of LBW and SGA.

In our population from the rural areas of Northwest China, 65.1% of the participants had anemia during pregnancy. Our prevalence of gestational anemia was much higher than that of Western countries such as the United States (5.7%), Canada (11.5%), and Germany (12.3%), and it was also higher than that of Asian countries such as Japan (14.8%) and Singapore (23.8%) [39]. Additionally, the prevalence was higher than that of native cities such as Beijing (19.3%), Guangzhou (38.8%), and Chengdu (23.9%) [40]. The potential reason for the high prevalence of anemia in our population is that a large apart of the participants were farmers, lower educated, and at low/middle-income levels, who were less likely to achieve a balanced diet and adequate nutrition intake during pregnancy [41], which further contributed to the poor nutritional status, and induced maternal anemia. At the other end of the spectrum, it is worth noting that about half of the participants had hemoglobin ≥ 130 during pregnancy, which indicated that the problems of low and high hemoglobin levels during pregnancy coexisted in the study population. 

In the present study, we found that maternal hemoglobin concentration in the third trimester was associated with neonatal birth weight in an inverted U-shaped curve and was associated with the risks of LBW and SGA in extended U-shaped curves. Women with severe anemia or hemoglobin > 130 g/L in the third trimester had significantly decreased neonatal birth weight and increased risks of LBW and SGA. Compared with previous studies [42,43], our results revealed a more complicated relationship between maternal hemoglobin and birth weight-related outcomes, in that both low and high hemoglobin concentration in the third trimester could have adverse effects on fetal weight growth. In our population, the relatively higher neonatal birth weight and lower risks of LBW and SGA were observed when hemoglobin concentration was in the range of 100 to 110 g/L, which implied that an appropriate hemoglobin level in the third trimester is beneficial to fetal weight growth, but the specific range still needs to be verified in future studies. 

Similar nonlinear associations between maternal hemoglobin concentration and birth weight-related outcomes were shown in several previous studies [10,11,26,44]. In a retrospective analysis in the North-West Thames region, Steer et al. reported that the maximum mean birth weight was achieved with the lowest maternal hemoglobin concentration in pregnancy of 85–95 g/l and the lowest incidence of LBW occurred with the lowest hemoglobin of 95–105 g/l. The results displayed that the lowest maternal hemoglobin during pregnancy might have an inverted U-shaped relationship with neonatal birth weight and a U-shaped relationship with the risk of LBW [10]. In a prospective cohort study in two South Asian countries (Pakistan and India), Ali et al. found that the mean neonatal birth weight is higher when the maternal hemoglobin during pregnancy was 110–129 g/L and it decreased when the hemoglobin was <110 g/L or >130 g/L. The study also showed a U-shaped association between maternal hemoglobin and the risk of LBW [26]. Jung et al.’s systematic review and meta-analysis reported an extended U-shaped association between maternal hemoglobin and the risk of SGA [45]. 

The timing of maternal hemoglobin measurement varied in previous studies, which was one of the causes of inconsistent results. We measured maternal hemoglobin concentration in each trimester and the significant associations of maternal hemoglobin with risks of LBW and SGA were observed only in the third trimester, which was partially consistent with the results of Young et al.’s systematic review and meta-analysis in 2019 [24]. This review reported that low maternal hemoglobin concentration was significantly associated with an increased risk of LBW in the first and third trimester, but it was not significantly associated with SGA in any trimester. Our study did not observe any significant association in the first trimester, which might be due to the relatively lower prevalence of anemia in the first trimester in our population and the different severity of anemia from the populations in previous studies. In addition, this review showed that the evidence for high maternal hemoglobin concentrations across pregnancy and LBW/SGA was limited. Our study provided new evidence that both low and high maternal hemoglobin concentrations in the third trimester was associated with increased risks of LBW/SGA, and to some extent, it filled in the gap of related research. One potential explanation for our results is as follows. Fetal weight growth velocity reaches a peak in the third trimester (around 35 weeks of gestation) accompanied by a sharp increase in fetal nutritional demand [46]. Severe anemia or a relatively-high hemoglobin concentration in this period may lead to extremely inadequate maternal nutrient supply to the fetus, which seriously affects fetal weight growth and increases the risks of LBW and SGA [44,47]. 

The possible mechanisms involved in the relationship between low or high hemoglobin concentrations and fetal weight growth are as follows. Severe anemia in the third trimester leads to a decline in the body’s oxygen supply capacity and poor placental development, which affects the maternal delivery of oxygen and nutrients to the fetus. These adverse effects further induce fetal chronic hypoxia and insufficient nutritional intake, which finally causes poor fetal weight growth and adverse birth outcomes such as LBW and SGA [26,47]. The harmful impacts of high maternal hemoglobin concentration on fetal growth may be attributed to the inadequate plasma volume expansion. A high hemoglobin concentration in the third trimester may indicate a failure in plasma volume expansion. Insufficient plasma volume expansion during pregnancy induces the increase of blood viscosity, and further leads to decrease in placental blood flow velocity and decline of placental nutrient delivery capacity, which finally affects fetal growth and development [18,44,48]. 

The plasma volume expansion is also an explanation for our results that better birth weight-related outcomes were obtained when maternal hemoglobin concentration in the third trimester was 100–110 g/L. The slightly lower hemoglobin concentrations in the third trimester may reflect an adequate plasma volume expansion and thus achieve the optimal nutrient transport from mother to fetus, which consequently promotes fetal growth and development [49]. Another possible explanation of the association of mild anemia and higher birth weight is that maternal absorbed iron may be preferentially transferred to the placenta and fetus during pregnancy, thus contributing to better fetal growth and development rather than to higher maternal iron stores, which may result in lower maternal hemoglobin concentration in the third trimester and higher neonatal birth weight [50].

To the best of our knowledge, the present study was the first prospective study that revealed nonlinear relationships between maternal hemoglobin concentration in the third trimester and birth weight-related outcomes in the Chinese population. We disclosed that both low and high hemoglobin concentrations could be harmful to fetal weight growth and increase the risks of LBW and SGA, and maternal hemoglobin > 130 g/L in the third trimester could be highly focused on, in addition to severe anemia, in maternal and child health care. Nonetheless, several limitations of the present study should be addressed. Firstly, this study was conducted in the rural areas of Northwest China, and thus the results mainly reflected the association between maternal hemoglobin during pregnancy and birth outcomes in women with disadvantaged socio-demographic conditions, which may limit the generalizability of the findings to other populations. However, it is noteworthy that, compared with women with disadvantaged socio-demographic status, those with advantaged socio-demographic status are more likely to have relatively higher hemoglobin concentrations during pregnancy, suggesting that the adverse impact of high hemoglobin in the third trimester on fetal weight growth should be paid more attention to in such populations. Secondly, although we carried out the analysis by controlling for some potential confounders, including socio-demographic and health-related factors, there were still some unobserved or unknown confounders that we could not fully investigate. For example, excessive maternal gestational weight gain was related to reduced risks of LBW and SGA [51]. Unfortunately, information about maternal weight gain during pregnancy was not available in the present study, and thus we could not control for its effect on the associations between maternal hemoglobin and birth weight-related outcomes. Finally, the present study only assessed the relationship between maternal hemoglobin and birth weight-related outcomes without evaluating the influences of maternal plasma volume expansion or iron status of mothers and infants, and thus the potential mechanisms involved could not be clarified. More in-depth and well-designed studies are recommended to explore the underlying mechanisms and to develop a more comprehensive and precise nutrition intervention strategy.

## 5. Conclusions

Maternal hemoglobin concentration in the third trimester had an inverted U-shaped association with neonatal birth weight, and had extended U-shaped associations with the risks of LBW and SGA. Both low and high hemoglobin concentrations in this period could be adverse to fetal weight growth and increase the risks of LBW and SGA, respectively. In addition to severe anemia, maternal hemoglobin > 130 g/L in the third trimester should be paid great attention to in the practice of maternal and child health care.

## Figures and Tables

**Table 1:** Maternal baseline characteristics and neonatal birth outcomes (*N*= 3748).

Characteristics	Mean ± SD or n (%)
Socio-demographic characteristics	
Age (years)	26.2 ± 4.1
<25	1369 (36.5)
25–34	2235 (59.6)
≥35	144 (3.8)
Education	
Junior high school or below	2040 (54.4)
Senior high school	1179 (31.5)
College or above	529 (14.1)
Farmers	3292 (87.8)
Per capita annual household income (RMB)	
Low (<5000)	872 (23.3)
Medium (5000–9999)	1519 (40.5)
High (≥10,000)	1357 (36.2)
Health-related characteristics	
Primipara	1914 (51.1)
Gestational age at enrolment (weeks)	14.4 ± 6.1
≤12	1580 (42.2)
>12	2168 (57.8)
Height (cm)	159.8 ± 4.8
Weight at enrolment (kg)	55.5 ± 8.11
BMI at enrolment (kg/m^2^)	
Underweight (<18.5)	408 (10.9)
Normal weight (18.5–23.9)	2662 (71.0)
Overweight (24.0–27.9)	553 (14.8)
Obesity (≥28.0)	125 (3.3)
More than five antenatal visits	1229 (32.8)
Micronutrient supplementation	
Folic acid	1363 (36.3)
Folic acid + iron	1130 (30.1)
Folic acid + vitamin B complex	1255 (33.4)
Birth outcomes	
Birth weight (g)	3233.4 ± 418.3
Gestational age at delivery (weeks)	39.7 ± 1.3
Gender, male	1936 (51.7)
LBW	99 (2.6)
SGA	501 (13.4)

LBW, low birth weight; SGA, small for gestational age.

**Table 2:** Maternal hemoglobin status in different trimesters of pregnancy.

Hemoglobin (g/L)	Mean ± SD or n (%)
First trimester	
Average hemoglobin concentration	123.2 ± 14.4
Anemia (<110)	624 (16.6)
Severe anemia (<70)	0 (0.0)
Moderate anemia (70–99)	203 (5.4)
Mild anemia (100–109)	421 (11.2)
Normal (110–129)	1883 (50.2)
Hemoglobin ≥ 130	1241 (33.1)
Second trimester	
Average hemoglobin concentration	115.6 ± 13.4
Anemia (<110)	1157 (30.9)
Severe anemia (<70)	0 (0.0)
Moderate anemia (70–99)	427 (11.4)
Mild anemia (100–109)	730 (19.5)
Normal (110–129)	2039 (54.4)
Hemoglobin ≥ 130	552 (14.7)
Third trimester	
Average hemoglobin concentration	110.8 ± 13.9
Anemia (<110)	1720 (45.9)
Severe anemia (<70)	12 (0.3)
Moderate anemia (70–99)	719 (19.2)
Mild anemia (100–109)	989 (26.4)
Normal (110–129)	1720 (45.9)
Hemoglobin ≥ 130	308 (8.2)

**Table 3:** Associations between maternal hemoglobin concentrations during pregnancy with neonatal birth weight, LBW, and SGA ^1^.

Outcomes	Hemoglobin (g/L)	Mean (SD) or n (%)	Unadjusted Model	Adjusted Model ^2^
		Mean (SD)	Changes (95% CI)	Changes (95% CI)
Birth weight	First trimester			
	<70	-	-	-
	70–99	3240.6 (415.7)	21.0 (−51.0, 92.9)	26.6 (−34.0, 87.2)
	100–109	3238.1 (418.0)	18.4 (−29.5, 66.2)	25.1 (−20.5, 70.7)
	110–129	3221.1 (410.1)	Ref.	Ref.
	≥130	3249.1 (430.8)	29.0 (1.7, 56.4)	26.5 (0.2, 52.8)
	Second trimester			
	<70	-	-	-
	70–99	3258.0 (426.1)	27.5 (−3.3, 58.3)	30.8 (−3.2, 64.8)
	100–109	3221.8 (412.1)	−9.3 (−47.9, 29.4)	−5.0 (−44.6, 34.7)
	110–129	3230.1 (424.8)	Ref.	Ref.
	≥130	3241.5 (395.4)	10.9 (−26.9, 48.6)	11.5 (−22.8, 45.7)
	Third trimester			
	<70	2988.3 (549.6)	−230.5 (−455.2, −5.7)	−216.3 (−426.7, −5.9)
	70–99	3253.3 (413.5)	35.9 (−0.2, 71.9)	45.8 (9.9, 81.7)
	100–109	3259.2 (440.5)	42.1 (5.4, 78.8)	46.5 (7.6, 85.3)
	110–129	3220.0 (409.0)	Ref.	Ref.
	≥130	3193.4 (419.7)	−29.2 (−72.5, 14.2)	−30.7 (−76.1, 14.7)
		n (%)	RR (95% CI)	RR (95% CI)
LBW	First trimester			
	<70	-	-	-
	70–99	4 (2.0)	0.67 (0.22, 2.06)	0.72 (0.24, 2.16)
	100–109	9 (2.1)	0.77 (0.39, 1.54)	0.80 (0.39, 1.63)
	110–129	54 (2.9)	Ref.	Ref.
	≥130	32 (2.6)	0.91 (0.61, 1.38)	0.85 (0.54, 1.33)
	Second trimester			
	<70	-	-	-
	70–99	12 (2.8)	0.95 (0.44, 2.02)	1.10 (0.52, 2.29)
	100–109	17 (2.3)	0.79 (0.47, 1.34)	0.90 (0.52, 1.55)
	110–129	59 (2.9)	Ref.	Ref.
	≥130	11 (2.0)	0.70 (0.35, 1.41)	0.53 (0.20, 1.37)
	Third trimester			
	<70	2 (16.7)	6.36 (2.07, 19.59)	7.47 (2.53, 22.08)
	70–99	21 (2.9)	1.10 (0.76, 1.61)	1.20 (0.76, 1.91)
	100–109	23 (2.3)	0.88 (0.57, 1.37)	0.94 (0.58, 1.51)
	110–129	42 (2.4)	Ref.	Ref.
	≥130	11 (3.6)	1.51 (0.64, 3.54)	1.41 (0.49, 4.03)
SGA	First trimester			
	<70	-	-	-
	70–99	27 (13.3)	0.97 (0.68, 1.39)	0.98 (0.67, 1.44)
	100–109	51 (12.2)	0.89 (0.65, 1.23)	0.91 (0.65, 1.29)
	110–129	256 (13.6)	Ref.	Ref.
	≥130	167 (13.5)	0.99 (0.82, 1.20)	0.99 (0.81, 1.21)
	Second trimester			
	<70	-	-	-
	70–99	56 (13.1)	0.94 (0.77, 1.16)	1.03 (0.83, 1.28)
	100–109	90 (12.4)	0.89 (0.75, 1.06)	0.91 (0.76, 1.09)
	110–129	285 (14.0)	Ref.	Ref.
	≥130	70 (12.7)	0.91 (0.74, 1.11)	0.95 (0.75, 1.20)
	Third trimester			
	<70	2 (16.7)	1.12 (0.31, 4.11)	1.32 (0.35, 4.98)
	70–99	91 (12.7)	0.86 (0.70, 1.05)	0.87 (0.70, 1.09)
	100–109	104 (10.5)	0.72 (0.61, 0.84)	0.73 (0.61, 0.87)
	110–129	252 (14.7)	Ref.	Ref.
	≥130	52 (16.9)	1.16 (0.88, 1.53)	1.16 (0.87, 1.55)

LBW, low birth weight; SGA, small for gestational age; Ref., reference, SD, standard deviation; RR, relative risk; CI, confident interval. ^1^ Generalized estimating equation models with random effect at the township level were used to estimate the changes (95% CI) for birth weight and RR (95% CI) for LBW/SGA according to maternal hemoglobin level during pregnancy. ^2^ The model was adjusted for socio-demographic characteristics (including maternal age, education, occupation, and per capita annual household income) and health-related characteristics (including parity, BMI at enrolment, gestational age at enrolment, number of antenatal visits, and micronutrient supplementation). When estimating the associations between maternal hemoglobin and birth weight/LBW, models were additionally adjusted for neonatal gender and gestational age at delivery.

## Supplementary Materials

The followings are available online at https://www.mdpi.com/article/10.3390/nu14040858/s1, Table S1: Comparison of maternal socio-demographic characteristics between women included and excluded in the study; Table S2: Associations between maternal hemoglobin concentration in the third trimester and neonatal birth weight in different subgroups; Table S3: Associations between maternal hemoglobin concentration in the third trimester and LBW/SGA in different subgroups.
