# Supplementary material for: Maternal Hemoglobin Concentrations and Birth Weight, Low Birth Weight (LBW), and Small for Gestational Age (SGA): Findings from a Prospective Study in Northwest China
Source: Nutrients. 2022 Feb 18;14(4):858. doi: 10.3390/nu14040858 (PMC8879779; doi:10.3390/nu14040858)
Supplement: Supplementary file 1 [file nutrients-14-00858-s001.zip › nutrients-1567362-supplementary.pdf]

Table S1. Comparison of maternal socio-demographic characteristics between women included and excluded in the study

Characteristics	Women included N=3748 n (%)	Women excluded N=635 n (%)	P [1]
Age (years)			0.593
< 25	1369 (36.5)	244 (38.4)	
25 - 34	2235 (59.6)	365 (57.5)	
≥ 35	144 (3.8)	26 (4.1)	
Education			0.118
Junior high school or below	2040 (54.4)	318 (50.1)	
Senior high school	1179 (31.5)	222 (35.0)	
College or above	529 (14.1)	95 (14.9)	
Occupation			0.388
Farmers	3292 (87.8)	550 (86.6)	
Others	456 (12.2)	85 (13.4)	
Per capita annual household income (RMB)			0.071
Low (< 5000)	872 (23.3)	139 (21.9)	
Medium (5000 – 9999)	1519 (40.5)	236 (37.2)	
High (≥ 10000)	1357 (36.2)	260 (40.9)	
Parity			0.451
Primipara	1914 (51.1)	314 (49.4)	
Multipara	1834 (48.9)	321 (50.6)	

[1] P values for the differences between groups were derived from $\chi 2$ tests or Fisher's exact test.

Table S2. Associations between maternal hemoglobin concentration in the third trimester and neonatal birth weight in different subgroups [1]

Subgroups	HGB<100 g/L			HGB≥100 g/L		
	N	Mean (SD)	Changes (95% CI) [2]	N	Mean (SD)	Changes (95% CI)
Overall	731	3254.8 (443.4)	2.4 (-0.5, 5.4)	3017	3228.2 (411.9)	-2.6 (-4.1, -1.0)
Age (years)						
< 25	248	3211.8 (407.9)	1.9 (-8.2, 12.1)	1121	3186.3 (408.2)	-2.5 (-4.4, -0.6)
25 - 34	458	3281.6 (454.6)	1.6 (-2.3, 5.4)	1777	3255.4 (410.3)	-2.6 (-4.2, -0.9)
≥ 35	25	3185.2 (527.5)	1.1 (-20.6, 22.8)	119	3215.1 (433.4)	-0.8 (-7.9, 6.2)
Education						
Junior high school or below	412	3242.2 (422.0)	2.0 (-2.6, 6.7)	1628	3233.8 (409.0)	-2.0 (-4.0, 0.1)
Senior high school	239	3263.8 (474.5)	0.8 (-6.1, 7.7)	940	3216.1 (415.7)	-2.6 (-5.2, -0.1)
College or above	80	3291.2 (452.9)	3.2 (-5.5, 11.8)	449	3232.8 (413.3)	-4.5 (-7.5, -1.6)
Per capita annual household income (RMB)						
Low (< 5000)	162	3270.4 (444.9)	3.6 (-9.3, 16.6)	710	3201.2 (429.8)	-1.4 (-4.2, 1.4)
Medium (5000 – 9999)	296	3244.4 (424.4)	1.5 (-4.1, 7.1)	1223	3232.2 (411.4)	-3.0 (-5.3, -0.7)
High (≥ 10000)	273	3256.3 (462.1)	3.0 (-3.2, 9.1)	1084	3241.2 (399.2)	-2.4 (-4.7, -0.1)
Parity						
Primipara	317	3253.3 (430.6)	0.6 (-4.8, 5.9)	1597	3199.8 (412.7)	-2.7 (-4.5, -0.8)
Multipara	414	3255.6 (452.5)	3.3 (-0.8, 7.3)	1420	3260.1 (408.3)	-2.3 (-4.3, -0.3)
Times of antenatal visits						
≤ 5	540	3228.4 (432.6)	1.3 (-2.6, 5.1)	1979	3225.9 (423.6)	-2.0 (-3.6, -0.5)
> 5	191	3328.7 (463.7)	4.4 (-3.0, 11.9)	1038	3232.4 (388.0)	-3.9 (-6.6, -1.3)
Micronutrient supplementation						
Folic acid	279	3250.7 (448.2)	3.5 (0.2, 6.8)	1084	3213.4 (405.3)	-3.3 (-5.1, -1.4)
Folic acid + iron	132	3289.5 (464.2)	6.5 (-8.8, 21.7)	998	3225.3 (411.0)	-1.1 (-3.1, 0.9)
Folic acid + vitamin B complex	320	3241.8 (429.3)	2.0 (-2.4, 6.5)	935	3248.8 (420.1)	-4.5 (-8.3, -0.7)

HGB, hemoglobin; SD, standard deviation; CI, confident interval.

[1] N=3748. Generalized estimating equation models with random effect at the township level were used to estimate the changes (95% CI) of birth weight in different subgroups according to materenal characteristics and hemoglobin level in the third trimester.

[2] The continuous variable of hemoglobin was used to estimate the changes (95% CI) of birth weight with per 1 g/L increase in the hemoglobin level in the third trimester. The models were adjusted for socio-demographic characteristics (including maternal age, education, occupation, and per capita annual household income), health-related characteristics (including parity, BMI at enrollment, gestational age at enrollment, number of antenatal visits, and micronutrient supplementation), as well as neonatal gender and gestational age at delivery, except for the variable stratified for the subgroup analysis.

Table S3. Associations between maternal hemoglobin concentration in the third trimester and LBW/SGA in different subgroups [1]

Subgroups	LBW				SGA			
	HGB<100 g/L		HGB≥100 g/L		HGB<100 g/L		HGB≥100 g/L	
	n (%)	RR (95% CI) [2]	n (%)	RR (95% CI)	n (%)	RR (95% CI)	n (%)	RR (95% CI)
Overall	23 (3.2)	0.58 (0.42, 0.79)	76 (2.5)	1.10 (0.81, 1.50)	93 (12.8)	0.81 (0.67, 0.98)	408 (13.6)	1.13 (1.04, 1.23)
Age (years)								
< 25	7 (2.8)	0.90 (0.16, 4.93)	33 (2.9)	0.94 (0.68, 1.30)	37 (14.9)	0.97 (0.59, 1.60)	183 (16.3)	1.15 (1.00, 1.31)
25 - 34	14 (3.1)	0.51 (0.35, 0.74)	39 (2.2)	1.24 (0.86, 1.79)	51 (11.1)	0.76 (0.61, 0.94)	204 (11.5)	1.10 (0.97, 1.25)
≥ 35	2 (8.0)	0.67 (0.26, 1.70)	4 (3.4)	1.19 (0.56, 2.52)	5 (20.0)	0.72 (0.33, 1.57)	21 (17.6)	1.16 (0.82, 1.64)
Education								
Junior high school or below	12 (3.0)	0.50 (0.41, 0.61)	39 (2.4)	1.23 (0.83, 1.81)	55 (13.3)	0.79 (0.66, 0.94)	231 (14.2)	1.15 (1.02, 1.30)
Senior high school	8 (3.3)	0.84 (0.34, 2.09)	28 (3.0)	0.97 (0.73, 1.29)	28 (11.7)	0.84 (0.53, 1.31)	117 (12.4)	1.06 (0.95, 1.18)
College or above	3 (3.8)	0.95 (0.47, 1.89)	9 (2.0)	1.11 (0.58, 2.11)	10 (12.5)	0.73 (0.30, 1.79)	60 (13.4)	1.11 (0.85, 1.45)
Per capita annual household income (RMB)								
Low (< 5000)	7 (4.3)	0.54 (0.38, 0.75)	25 (3.5)	1.26 (0.82, 1.92)	25 (15.4)	0.56 (0.48, 0.66)	125 (17.6)	1.04 (0.87, 1.24)
Medium (5000 – 9999)	5 (1.7)	0.35 (0.21, 0.58)	28 (2.3)	0.92 (0.59, 1.44)	35 (11.8)	0.96 (0.67, 1.39)	150 (12.3)	1.23 (1.09, 1.38)
High (≥ 10000)	11 (4.0)	0.94 (0.49, 1.81)	23 (2.1)	1.20 (0.95, 1.52)	33 (12.0)	0.88 (0.59, 1.31)	133 (12.3)	1.10 (0.96, 1.26)
Parity								
Primipara	7 (2.2)	0.99 (0.53, 1.87)	51 (3.2)	0.87 (0.67, 1.13)	48 (15.1)	0.88 (0.63, 1.23)	252 (15.8)	1.05 (0.95, 1.15)
Multipara	16 (3.9)	0.52 (0.42, 0.65)	24 (1.7)	1.68 (1.11, 2.55)	45 (10.9)	0.81 (0.59, 1.12)	156 (11.0)	1.30 (1.10, 1.53)
Times of antenatal visits								
≤ 5	19 (3.5)	0.70 (0.49, 1.00)	55 (2.8)	0.91 (0.64, 1.31)	73 (13.6)	0.84 (0.65, 1.08)	287 (14.5)	1.12 (1.03, 1.22)
> 5	4 (2.1)	0.43 (0.34, 0.54)	21 (2.0)	1.88 (1.25, 2.82)	20 (10.5)	0.98 (0.66, 1.45)	121 (11.7)	1.17 (1.04, 1.32)
Micronutrient supplementation								
Folic acid	8 (2.9)	0.47 (0.35, 0.64)	29 (2.7)	0.91 (0.59, 1.41)	36 (13.1)	0.63 (0.59, 0.68)	145 (13.4)	1.22 (1.11, 1.33)

Folic acid + iron	4 (3.0)	0.88 (0.40, 1.99)	30 (3.0)	1.22 (0.71, 2.10)	19 (12.9)	0.92 (0.65, 1.32)	139 (13.9)	1.03 (0.89, 1.19)
Folic acid + vitamin B complex	11 (3.4)	0.62 (0.34, 1.14)	17 (1.8)	1.19 (0.77, 1.83)	38 (12.4)	0.84 (0.60, 1.18)	124 (13.3)	1.16 (1.01, 1.34)

LBW, low birth weight; SGA, small for gestational age; HGB, hemoglobin; RR, relative risk; CI, confident interval.

[1] N=3748. Generalized estimating equation models with random effect at the township level were used to estimate the RR (95% CI) of LBW/SGA in different subgroups according to materenal characteristics and hemoglobin level in the third trimester.

[2] The continuous variable of hemoglobin was used to estimate the RR (95% CI) of LBW/SGA with per 10 g/L increase in the hemoglobin level in the third trimester. The models were adjusted for socio-demographic characteristics (including maternal age, education, occupation, and per capita annual household income), health-related characteristics (including parity, BMI at enrollment, gestational age at enrollment, number of antenatal visits, and micronutrient supplementation), as well as neonatal gender and gestational age at delivery (only in LBW models), except for the variable stratified for the subgroup analysis.
